# Genotypic and phenotypic features of dyslipidemia in a sample of pediatric patients in China

**DOI:** 10.1186/s12887-023-03952-z

**Published:** 2023-03-29

**Authors:** Qianwen Zhang, Guoying Chang, Yijun Tang, Shili Gu, Yu Ding, Yao Chen, Yirou Wang, Shijian Liu, Jian Wang, Xiumin Wang

**Affiliations:** 1 Department of Endocrinology and Metabolism, Shanghai Children’s Medical Center, School of Medicine, Shanghai Jiao Tong University, Shanghai, China; 2 Department of Clinical Epidemiology and Biostatistics, Shanghai Children’s Medical Center, School of Medicine, Shanghai Jiao Tong University, Shanghai, China; 3 Department of Medical Genetics and Molecular Diagnostic Laboratory, Shanghai Children’s Medical Center, School of Medicine, Shanghai Jiao Tong University, Shanghai, China; 4 Center for Brain Science, Shanghai Children’s Medical Center, School of Medicine, Shanghai Jiao Tong University, Shanghai, China

**Keywords:** Next-generation sequencing, Tendon xanthomas, Monogenic hypercholesterolemia

## Abstract

**Background:**

Dyslipidemia, especially hypercholesterolemia is of significant clinical interest. Precise diagnosis is not paid enough attention to about the management of pediatric patients with hypercholesterolemia, which is especially apparent in China. Given this, we designed this study to confirm the specific molecular defects associated with hypercholesterolemia using whole-exome sequencing (WES) to be helpful for precise diagnosis and treatment.

**Methods:**

Pediatric patients were enrolled using specific criteria and their clinical information were recorded for later evaluation in conjunction with the WES completed for each of these patients.

**Results:**

Our criteria allowed for the initial enrollment of 35 patients, 30 of whom (aged 1.02–12.99 years) underwent successful genetic sequencing and clinical investment. Positive results were obtained in 63.33% (19/30) of these patients. We identified 25 variants in 30 pediatric patients with persistent hypercholesterolemia, seven of them were novel and variants in *LDLR* and *ABCG5/ABCG8* ranks first and second, respectively. Further analysis revealed that the levels of total cholesterol (TC), low-density lipoprotein cholesterol (LDL-C), apolipoprotein B (ApoB) and lipoprotein (a) were higher in patients with positive genetic results.

**Conclusion:**

Our study enriched the genetic and phenotypic spectra for hypercholesterolemia in young patients. Genetic testing is important for the prognostics and treatment of pediatric patients. Heterozygous *ABCG5/8* variants may be underestimated in pediatric patients with hypercholesterolemia.

**Supplementary Information:**

The online version contains supplementary material available at 10.1186/s12887-023-03952-z.

## Background

Dyslipidemia is rapidly increasing in both children and adolescents, posing a threat to their health. Hypercholesterolemia, especially elevated low-density lipoprotein cholesterol (LDL-C) levels, are usually of specific focus and are a recognized risk factor for premature atherosclerotic cardiovascular disease (ASCVD) [1]. Dyslipidemia may occur for several reasons, including genetic and nongenetic factors. In pediatric patients especially for those with young age and normal weight, genetic factors probably contribute more on dyslipidemia.

Precise diagnosis is much more important for children with genetic testing, both in prognostics and treatment, comparing with that for adults. Identifying monogenic hypercholesterolemia in adult patients could be ignored as it has limited influence on the treatment [2]. Statins is the primary pharmacotherapy used to lower lipid. For the most adult patients in whom statins are indicated, the benefits outweigh the risks [3]. And ezetimibe is usually recommended to be used combined with statins in patients who have not been able to achieve 50% reduction in LDL-C level. However, treatment of pediatric dyslipidemia begins with lifestyle modifications, but primary genetic dyslipidemias may require medications [4]. Drug use in pediatric patients have strict indications and many lipid-lowering drugs were only recommended to be used in pediatric patients with homozygous FH or with elder age [4]. The use of combined drugs is even more difficult in the clinic considering the side effects and indications. Furthermore, a recent study found an association between receiving a genetic diagnosis of FH and willingness to be treated with medication, suggesting genetic diagnosis may be useful for cardiovascular prevention in children [5].

Precise diagnosis may be facilitated by the advent of next-generation sequencing (NGS) technologies, which have facilitated the identification of several specific genes for this condition including *LDLR, PCSK9, APOB, STAP1,* and *LDLRAP1* [6]. More than 25 genes have been identified in patients with dyslipidemia [7]. However, sequencing for these genes can only explain part of the patients [8] and most of studies have focused on some specific genes linked to familiar hypercholesterolemia, such as *LDLR*, *APOB,* and *PCSK9*, using gene panels instead of NGS, which may underestimate the proportion of monogenic dyslipidemia/hypercholesterolemia [9]. The genetic reason for dyslipidemia is often unclear and rarely analyzed in pediatric patients [10, 11]. In addition, if physical findings are identified in a child, rarer dyslipidemias should be considered [4].

Therefore, this study was designed to confirm the molecular defects of hypercholesterolemia in pediatric patients using whole-exome sequencing (WES) rather than gene panels of FH, basing on a single-center group of children and adolescents. We collected samples from 30 pediatric patients with hypercholesterolemia and performed WES, allowing us to link the genetic and phenotypic data of 63.33% of these patients. We also evaluated the differences in lipid levels and demographic data between patients with the positive genetic results and patients with negative genetic results. It is our hope that these results will help to expand the genetic spectrum for monogenic dyslipidemia and be beneficial to precise diagnosis and treatment.

## Methods

### Patients

This study was designed as a single-center retrospective evaluation of pediatric patients (aged < 18 years) with dyslipidemia in the Department of Endocrinology and Metabolism in Shanghai Children’s Medical Center (including patients in in-patient and out-patient care) between 2015 and 2021 basing on electronic medical record system. Only those patients meeting one of the following criteria were included: 1. Clinical diagnosis of persistent hypercholesterolemia; 2. Clinical diagnosis of premature ASCVD; 3. Presence of tendon xanthomas; 4. Hypercholesterolemia with a family history of hypercholesterolemia or premature ASCVD. Persistent hypercholesterolemia was defined as an LDL-C level of ≥3.60 mmol/L (140.00 mg/dL) one two separate occasions obtained at least three months apart according to the guidelines recommended by both the Japanese and Chinese medical authorities [2, 6]. Patients were excluded if they had apparent inducing factors or other conditions such as diabetic ketoacidosis, anorexia, malnutrition, acute pancreatitis, or severe liver/kidney disease, which might result in secondary hypercholesterolemia.

Ethical approval for this study was obtained from the ethics committee of Shanghai Children’s Medical Center. Written informed consent was obtained from all the participants or their guardians before WES was performed.

### Clinical assessment and laboratory investigation

Physical examination was performed including height, weight, and special reference to the presence of tendon xanthomas at the time of diagnosis. At the same time, blood samples were collected in ethylenediaminetetraacetic acid-containing-containing tubes early in the morning after an overnight fast and total cholesterol (TC), LDL-C, high-density lipoprotein cholesterol (HDL-C), apolipoprotein A1 (ApoA1), apolipoprotein B (ApoB), and triglycerides (TG) were evaluated with chemiluminescent method in the absence of any lipid-lowering therapy. Clinical information, including a history of diabetes mellitus, hypertension, ASCVD, and lipid-lowering treatment, and family history of dyslipidemia, was confirmed at the time of patients screening for eligibility by telephone.

### Genetic sequencing

Peripheral blood samples were collected from the patients and their parents after informed consent was obtained. WES was performed on these patients as mentioned before [12, 13]. A QIAamp DNA Blood Mini kit® (Qiagen GmbH, Hilden, Germany) was used to isolate genomic DNA. Library was established with an Agilent SureSelect Target Enrichment system (Agilent Technologies, Inc., Santa Clara, CA, USA). And the system of Illumina HiSeq 2000 (Illumina, Inc.) and an Illumina cBot (Illumina Inc., San Diego, CA, USA) were used to sequence and generate clusters. All variants detected were filtered and annotated by Ingenuity Variant Analysis (Ingenuity Systems, Redwood City, CA, USA). Finally, Sanger sequencing was used to confirm the variants detected by WES comparing to the individuals’ parents. The potential pathogenicity of the missense variant was evaluated using three in silico prediction methods: SIFT (http://sift.jcvi.org/), PolyPhen-2 (http://genetics.bwh.harvard.edu/pph2/), and MutationTaster (http://www.mutationtaster.org/ChrPos.html).

### Statistical analysis

Quantitative data showed as mean ± Standard Deviation (SD) and Shapiro-Wilk test was used to test the distribution. Then, comparisons were performed by nonparametric tests or unpaired t-test where appropriate. Qualitative data are expressed as frequency (%) and compared using Chi-squared test of Fisher test. SPSS 25.0 (Statistical Package for the Social Sciences Inc., Chicago, IL, USA) was used for statistical analysis. *P* < 0.05 was considered statistical significance with two-sides.

## Results

### Demographic data

**Fig. 1:**
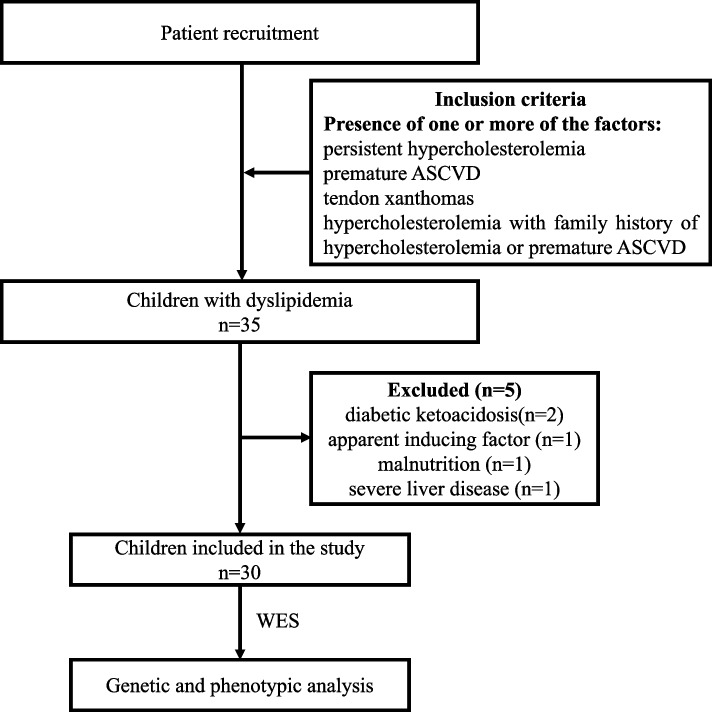
Flowchart of patient recruitment and variants discovery approach. ASCVD, atherosclerotic cardiovascular disease; MH, monogenic hypercholesterolemia; WES, whole-exome sequencing

We initially recruited 35 patients who were then subject to carefully check of medical records, resulting in the exclusion of five more patients due to severe diabetic ketoacidosis, apparent inducing factors (high fat diet), malnutrition, and severe liver disease. This left us with 30 patients (13 males and 17 females), all of whom were from non-consanguineous families (Fig. 1). Demographic data is summarized in Table 1. Their average age was found to be 6.42 ± 3.20 years. Six patients presented with tendon xanthomas and 13 patients had a family history of hypercholesterolemia. Detailed data for each of these patients is summarized in Supplementary Table S1.

**Table 1:** Demographic data of the patients in this study

**Subject**	
Number	30
Gender	13 M/17F
Age (mean ± SD)	6.42 ± 3.20
Tendon xanthomas, n/N	6/30
Family history, n/N	13/25
BMI (mean ± SD)	18.09 ± 4.70
**Laboratory result (mean ± SD)**	
Total cholesterol (0–5.2 mmol/L)	7.74 ± 2.11
Triglyceride (0–1.7 mmol/L)	1.46 ± 0.95
HDL cholesterol (0.9–1.68 mmol/L)	1.38 ± 0.39
LDL cholesterol (< 3.6 mmol/L)	5.68 ± 2.16
Apolipoprotein A1 (1.04–2.02 g/L)	1.35 ± 0.28
Apolipoprotein B (0.66–1.33 g/L)	1.56 ± 0.54
Lipoprotein (0-75 nmol/L)	52.24 ± 58.89
**Genes related**	*LDLR, LIPC, ABCG5, ABCG8, LPL, CETP*

### Genetic results

The candidate variants were firstly screened by a minor allele frequency < 1%. Then, they were analyzed while hypercholesterolemia was selected as the main filtering symptom. For missense variants, potential pathogenicity was evaluated using three in silico prediction methods. Finally, all variants detected were classified according to the guideline recommended by The American College of Medical Genetics and Genomics (ACMG).

**Fig. 2:**
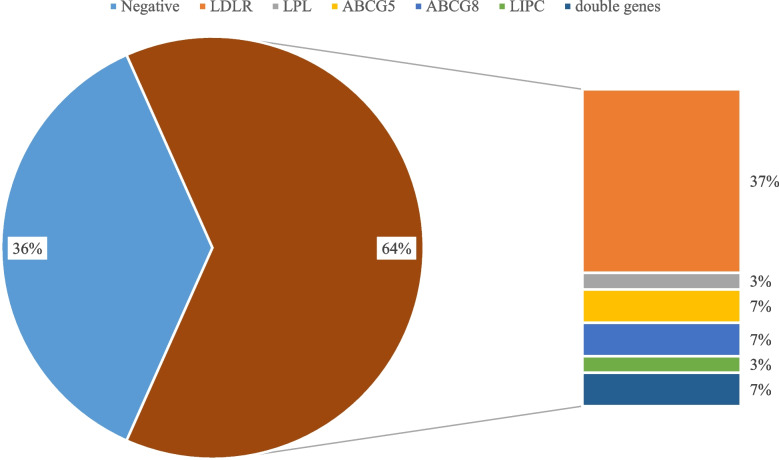
Spectrum of the genes of patients with hypercholesterolemia

We identified 25 variants in these patients, seven of which were novel (Tables 1 and 2, and Supplementary Table S1). Among the 30 patients, 19 (63.33%) were identified positive genetic results and single gene variants were confirmed in 17 patients. The other two patients were identified variants of two different genes (Fig. 2 and Table 1). Evaluation revealed that there were *LDLR* variations in 13 patients, making it the most common mutation in this study which is likely explained by the high carrier rate in this population. Other variants included mutations in *ABCG5, ABCG8, LIPC, LPL,* and *CETP* (Fig. 2).

**Table 2:** Variants identified in this study

Gene	Refseq	DNA change	Protein change	MAF (gnomAD)	Mutation taster	PolyPhen-2	SIFT	ACMG classification
*ABCG5*	NM_022436.2	c.1673_1677del	p.Pro558Glnfs*14	0.00%	/	/	/	LP
*ABCG5*	NM_022436.2	c.1336C > T	p.Arg446*	0.02%	/	/	/	P
*ABCG5*	NM_022436.2	c.1762 + 1G > A	p.?	0.00%	/	/	/	P
***ABCG5***	**NM_022436.2**	**c.64C > T**	p.Gln22*	**0.00%**	**/**	**/**	**/**	**LP**
*ABCG8*	NM_022437.2	c.788G > A	p.Arg263Gln	0.01%	Disease-causing	Probably damaging	Damaging	P
***ABCG8***	**NM_022437.2**	**c.1938_1939delins**	p.Val647Serfs*16	**0.00%**	**/**	**/**	**/**	**P**
*ABCG8*	NM_022437.2	c.1256_1257delins	p.Ile419Lys	0.00%	/	/	/	VUS
***CETP***	**NM_000078.2**	**c.1103del**	p.Pro368Hisfs*9	**0.00%**	**/**	**/**	**/**	**LP**
*LDLR*	NM_000527.4	c.2389G > A	p.Val797Met	0.00%	Disease-causing	Probably damaging	Tolerable	P
*LDLR*	NM_000527.4	c.1448G > A	p.Trp483*	0.00%	/	/	/	P
*LDLR*	NM_000527.4	c.682G > T	p.Glu228*	0.00%	/	/	/	P
***LDLR***	**NM_000527.4**	**c.1338del**	p.Ser447Profs*4	**0.00%**	**/**	**/**	**/**	**P**
*LDLR*	NM_000527.4	c.380 T > A	p.Val127Asp	0.00%	Disease-causing	Probably damaging	Damaging	LP
*LDLR*	NM_000527.4	c.599 T > G	p.Phe200Cys	0.00%	Polymorphism	Probably damaging	Tolerable	LP
*LDLR*	NM_000527.4	c.1285G > A	p.Val429Met	0.00%	Disease-causing	Probably damaging	Damaging	P
*LDLR*	NM_000527.4	c.2389G > A	p.Val797Met	0.00%	Disease-causing	Probably damaging	Tolerable	P
*LDLR*	NM_000527.4	c.327C > A	p.Cys109*	0.00%	/	/	/	P
*LDLR*	NM_000527.4	c.817 + 1G > A	p.?	0.00%	/	/	/	P
***LDLR***	**NM_000527.4**	**c.1298A > T**	**p.Asp433Val**	**0.00%**	**Disease-causing**	**Probably damaging**	**Damaging**	**LP**
*LDLR*	NM_000527.4	c.1060 + 10G > A	p.?	0.00%	/	/	/	VUS
*LDLR*	NM_000527.4	c.1187-10G > A	p.?	0.00%	/	/	/	P
*LDLR*	NM_000527.4	c.905G > T	p.Cys302Phe	0.00%	Disease-causing	Probably damaging	Damaging	LP
***LIPC***	**NM_000236.2**	**c.748 T > C**	**p.Phe250Leu**	**0.00%**	**Disease-causing**	**Probably damaging**	**Damaging**	**LP**
***LIPC***	**NM_000236.2**	**c.1052-1G > C**	**p.?**	**0.00%**	**/**	**/**	**/**	**P**
*LPL*	NM_000237.3	c.1187A > T	p.Glu396Val	0.00%	Disease-causing	Probably damaging	Damaging	LP

*MAF* Minor Allele Frequency. Bold values, novel variants

### Subgroup analysis

Next, we wanted to determine if there were any distinct differences between different subgroups. Firstly, we analyzed the patients with and without positive genetic results. To this end we analyzed the age, gender, incidence of tendon xanthomas, BMI, family history of hypercholesterolemia, and blood lipid levels in each of these groups of patients (Table 3) and found that patients with positive genetic results presented with elevated TC, LDL-C, ApoB, and Lp (a). In addition, 86.67% of the patients in the positive genetic result group had a family history of hypercholesterolemia, while no patient in the other group had a family history of this condition. No significant differences in other aspects of these patients including age, gender, incidence of tendon xanthomas, BMI, and levels of TG, HDL-C, and ApoA1were identified when comparing these two groups. Next, we did similar analysis between patients with LDLR heterozygous and ABCG5/8 heterozygous. No significant differences were found between these two groups.

**Table 3:** Comparison of patients grouped by WES result and gene

Subject	Grouped by WES			Grouped by Gene		
	WES-positive *N* = 19	WES-negative *N* = 11	*P* value	*LDLR* heterozygous *N* = 10	*ABCG5/8* heterozygous *N* = 3	*P* value
Age (mean ± SD, y)	7.29 ± 3.08	4.95 ± 2.99	0.053 ^a^	7.96 ± 3.07	4.38 ± 1.84	0.087 ^a^
Gender (M/F)	8/11	5/6	1.000 ^b^	6/4	2/1	1.000 ^b^
Tendon xanthomas, n/N (%)	5/18 (27.78)	1/11 (9.09)	0.362 ^b^	1/10 (10.00)	1/3 (33.33)	0.423 ^b^
Family history, n/N (%)	13/15 (86.67)	0/10 (0.00)	< 0.001 ***^b^	7/7 (100)	2/3 (66.66)	0.300 ^b^
BMI (mean ± SD, kg/m2)	18.04 ± 3.99	18.13 ± 5.76	0.666 ^c^	17.05 ± 3.02	15.47 ± 0.80	0.409 ^a^
TC (mean ± SD, mmol/L)	8.56 ± 2.12	6.33 ± 1.15	< 0.001 ***^c^	8.31 ± 1.11	8.01 ± 0.92	0.678 ^a^
TG (mean ± SD, mmol/L)	1.44 ± 0.76	1.50 ± 1.25	0.553 ^c^	1.49 ± 0.71	0.85 ± 0.25	0.077 ^c^
HDL-C (mean ± SD, mmol/L)	1.41 ± 0.40	1.34 ± 0.38	0.889 ^a^	1.41 ± 0.43	1.62 ± 0.34	0.458 ^a^
LDL-C (mean ± SD, mmol/L)	6.48 ± 2.27	4.30 ± 1.01	0.002 **^c^	6.22 ± 1.05	6.00 ± 0.65	0.746 ^a^
ApoA1(mean ± SD, g/L)	1.31 ± 0.28	1.42 ± 0.28	0.553 ^c^	1.32 ± 0.25	1.47 ± 0.17	0.335 ^a^
ApoB (mean ± SD, g/L)	1.74 ± 0.57	1.26 ± 0.32	0.002 **^c^	1.61 ± 0.24	1.60 ± 0.12	0.939 ^a^
Lp (a) (mean ± SD, nmol/L)	67.01 ± 63.86	26.74 ± 39.85	0.018 *^c^	78.5 ± 70.98	17.13 ± 16.40	0.177 ^a^

*WES* Whole-exome sequencing, *F* Female, *M* Male, *SD* Standard deviation, *BMI* Body mass index, *TC* Total cholesterol, *TG* Triglyceride, *HDL-C* High-density lipoprotein cholesterol, *LDL-C* Low-density lipoprotein cholesterol, *ApoA1* Apolipoprotein A1, *ApoB* Apolipoprotein B, *Lp (a)* Lipoprotein (a)

^a^*P* value by the independent samples t-test with equal variance

^b^*P*-value by the Fisher exact test

^c^*P* value by the Mann-Whitney U test

* *P* < 0.05, ** *P* < 0.01, *** *P* < 0.001

## Discussion

This study describes both the genotypic and phenotypic data of 30 pediatric patients with dyslipidemia, using WES to minimize bias in genetic selection. Most of the patients evaluated in our study were below ten years of age. To the best of our known, this is one of the largest studies of pediatric hypercholesterolemia in China.

WES have advantages in diagnosing dyslipidemia in pediatric patients comparing with gene panels of FH. Gene panels is cost-effective but probably lead to miss diagnosis in some patients. FH has received more attention than other types of dyslipidemia, and scientists have emphasized the importance of several genes in this condition, with many of the available drugs designed to treat this specific condition [6, 14]. The most common pathogenic mutations in FH appear within the *LDLR* gene [15], which was further validated by our study. Many studies sequenced specific genes for patients with clinically suspected FH [16]. Genes encoding *LDLR, APOB, PCSK9* are recommended to include in the genetic testing [17]. Minicocci et,al. sequenced *LDLR, APOB, PCSK9* in 78 children and adolescents with clinically diagnosed FH and identified FH-causing mutations in 50% of them [9]. Comparing with their results, we identified positive genetic results in 63.33% of the pediatric patients. 11 patients were identified *LDLR* variants, and eight patients were identified other pathogenic genes like *ABCG5/8, LPL, LIPC*, and *CETP*, which are usually not included in FH gene panels. WES could be an effective complement to FH gene panels, especially in patients with negative results of FH gene panels. Considering the difficulty of general use of WES as a means of screening in nationally, we recommended to sequence more genes in pediatric patients with negative genetic results.

Some of the genes identified in this study are not common in patients with hypercholesterolemia. One of them is lipoprotein lipase (LPL), which is responsible for the intravascular hydrolysis of the TG in TG-rich lipoproteins. Homozygous or compound heterozygous variants in *LPL* gene could result in the accumulation of TG-rich lipoproteins while heterozygotes for *LPL* mutations present with variable plasma TG levels, ranging from normal to very high levels (> 10 mmol/L) and decreased levels of HDL-C [18]. The patient in our study with mutations in this gene presented with mildly elevated levels of TC, TG, and LDL-C and had no tendon xanthomas or abnormal HDL-C levels. Mutations in *LIPC* and *CETP* are both associated with reduced HDL-C levels and hyperalphalipoproteinemiam [19]. However, the two patients carrying variants of *LIPC* or *CETP* did not present with elevated ApoA1 or decreased HDL-C. They only presented with abnormal levels of TC and LDL-C levels.

Pediatric patients with hypercholesterolemia could be resulted by heterozygous *ABCG5/8* variants. Our study shows that *ABCG5/8* could be underestimated in pediatric patients with hypercholesterolemia and NGS has an advantage in diagnosing sitosterolemia or carriers of *ABCG5/8* gene comparing to gene panels of FH. *ABCG5/8* is the pathogenic genes associated with sitosterolemia, characterized by increasing levels of plant sterols [20]. In our study, 26.32% (5/19) of the patients were identified variants in *ABCG5/8*. 50.00% (3/6) of the patients with xanthomas were confirmed having at least one *ABCG5/8* variant, indicating that xanthomas are probably an indicator of ABCG5/8 variant. Mauricio, et,al. found 3.10% of the patients were diagnosed sitosterolemia, through sequencing *ABCG5/8* genes in 260 patients with clinical diagnosed FH and negative genetic results [21]. However, they did not analyze carrier rate of *ABCG5/8* gene [21]. Recent studies showed that carriers of *ABCG5/8* gene present with elevated phytosterol levels and are at increased risk of CAD [22]. Given the difficulties associated with serum sitosterol testing in China and difference in treatment between sitosterolemia and other types of hypercholesterolemia in children, NGS has become much more important in the diagnosis of these patients, especially for those who presented with xanthomas. However, it is still controversial that if patients with heterozygous *ABCG5/8* variants should be treated with medicine. And these patients should be followed-up and monitored regularly.

Compared to the patients with negative genetic results, patients with positive genetic results had significantly greater ApoB and Lp (a) levels (Table 3). ApoB, as an essential constituent of very-low-density lipoprotein and its metabolites intermediate density lipoproteins and LDLs, as well as chylomicrons and their remnants, and is crucial for the maintenance of the structural stability of various lipoproteins [23, 24]. Strong evidence shows that ApoB is a more accurate indicator of cardiovascular risk than either TC or LDL-C [24]. Our study indicates that ApoB is also a potential biomarker or therapeutic target for monogenic hypercholesterolemia.

The incidence rate of tendon xanthomas is up to 20.00% in this study, which is probably resulted by Berkson’s bias. Among the six patients presenting with tendon xanthomas, five had positive genetic results, with a diagnosis rate of 83.33%, while the diagnosis rate in patients without tendon xanthomas was 58.33% (14/24). A meta-analysis showed that age, male gender, LDL-C and TG level were associated with the presence of xanthomas and that this condition indicates an increased risk of CVD [25]. Similar studies are rare in pediatric patients. Our result suggests that xanthomas remain a strong indicator for monogenic hypercholesteremia.

However, there are also some limitations in this study. First of all, the numbers of patients enrolled in this study is small. Lack of widely screening of FH in China could partly explain the phenomenon. Children’s parents could be not aware of the exact family history of hypercholesterolemia. Also, we did not list other symptoms of hypercholesterolemia like corneal arcus as an inclusion criterion. These factors could result in that children with milder profiles may not be captured in this study. Secondly, lack of control group. We didn’t analyze rate of carries with NGS in healthy patients given the economic factors.

## Conclusions

In conclusion, we reported genetic and phenotypic description of 30 Chinese pediatric patients presenting with hypercholesterolemia/tendon xanthomas. Genetic testing with WES is important for the prognostics and treatment of pediatric patients with hypercholesterolemia. In addition, our results suggest that heterozygous ABCG5/8 variants may be underestimated in pediatric patients with hypercholesterolemia, especially for those presented with xanthomas.

## Supplementary Information

**Additional file 1.**

## Data Availability

All novel variants have been submitted to the NCBI ClinVar database whose accession number is SCV002570177 (https://www.ncbi.nlm.nih.gov/clinvar/variation/1704642/?oq=SCV002570177). The raw datasets generated and/or analyzed during the current study are available from the corresponding author upon reasonable request.

## Abbreviations

LDL-C: Low-density lipoprotein cholesterol
ASCVD: Atherosclerotic cardiovascular disease
FH: Familial hypercholesterolemia
NGS: Next-generation sequencing
WES: Whole-exome sequencing
TC: Total cholesterol
HDL-C: High-density lipoprotein cholesterol
ApoA1: Apolipoprotein A1
ApoB: Apolipoprotein B
TG: Triglycerides
SD: Standard deviation
MH: Monogenic hypercholesterolemia
Lp (a): Lipoprotein (a)
BMI: Body mass index
ACMG: The American College of Medical Genetics and Genomics
F: Female
M: Male
