# Supplementary material for: Genotypic and phenotypic features of dyslipidemia in a sample of pediatric patients in China
Source: BMC Pediatr. 2023 Mar 29;23:138. doi: 10.1186/s12887-023-03952-z (PMC10053209; doi:10.1186/s12887-023-03952-z)
Supplement: Supplementary file 1 — Additional file 1. [file 12887_2023_3952_MOESM1_ESM.docx]

Table S1. Detailed information of the patients in this study.

| Patient | Gender | Age | tendon xanthomas | Family history | Height | Weight | BMI | TC (0-5.2mmol/L) | TG(0-1.7mmol/L) | HDL-C(0.9-1.68mmol/L) | LDL-C(<3.6mmol/L) | ApoA1 (1.04-2.02g/L) | ApoB (0.66-1.33g/L) | Lp (a) (0-75nmol/L) | Gene ^†^ | Other Findings |
| --- | --- | --- | --- | --- | --- | --- | --- | --- | --- | --- | --- | --- | --- | --- | --- | --- |
| 1 | M | 3.99 | N | N | 94.00 | 15.00 | 16.98 | 5.69 | 0.83 | 1.68 | 3.63 | 1.71 | 0.98 | 17.6 | - | - |
| 2 | M | 1.02 | Y | NA | NA | NA | NA | 5.68 | 0.56 | 1.46 | 3.97 | 1.37 | 1.07 | 0.2 | - | - |
| 3 | F | 6.83 | N | N | 112.5 | 18.00 | 14.35 | 5.25 | 0.78 | 1.14 | 3.76 | 1.38 | 0.97 | 1.6 | - | - |
| 4 | F | 4.29 | N | N | 107.50 | 17.90 | 15.49 | 6.43 | 0.91 | 2.03 | 3.99 | 1.96 | 1.18 | 8.3 | - | inequality length of lower extremity |
| 5 | M | 4.48 | N | N | 98.20 | 16.30 | 16.90 | 5.64 | 0.85 | 1.55 | 3.7 | 1.77 | 1.06 | 117.1 | - | - |
| 6 | F | 5.10 | N | N | 114.00 | 36.60 | 28.16 | 5.86 | 3.33 | 0.68 | 3.67 | 1.25 | 1.54 | 6.9 | - | acanthosis nigricans |
| 7 | M | 12.90 | N | N | 168.60 | 83.60 | 29.41 | 7.45 | 4.44 | 0.95 | 4.48 | 1.08 | 1.59 | 21.6 | - | - |
| 8 | M | 3.30 | N | N | 91.00 | 14.50 | 17.51 | 5.54 | 0.83 | 1.46 | 3.7 | 1.29 | 0.9 | 2 | - | - |
| 9 | F | 4.97 | N | N | 111.60 | 18.00 | 14.45 | 5.71 | 0.82 | 1.31 | 4.03 | 1.24 | 1.09 | 93.2 | - | - |
| 10 | F | 3.98 | N | N | 93.50 | 11.75 | 13.44 | 7.34 | 1.37 | 0.99 | 5.73 | 1.08 | 1.58 | 20.4 | - | Intrauterine growth restriction |
| 11 | F | 8.54 | N | Y | 126.50 | 25.70 | 16.06 | 8.44 | 2.68 | 1.21 | 6.01 | 1.31 | 1.6 | 45.7 | LDLR(c.2389G>A) | - |
| 12 | F | 3.91 | N | NA | 108.00 | 16.00 | 13.72 | 9.53 | 0.88 | 1.6 | 7.53 | 1.38 | 1.85 | 7.8 | LDLR(c.1448G>A) | - |
| 13 | F | 3.64 | N | N | 101.00 | 15.00 | 14.70 | 9.02 | 1.76 | 1.48 | 6.74 | 1.44 | 1.86 | 5.2 | - | - |
| 14 | F | 6.57 | Y | Y | 145.00 | 35.00 | 16.65 | 7.77 | 0.98 | 1.42 | 5.9 | 1.27 | 1.59 | 13.6 | LDLR(c.682G>T) | - |
| 15 | F | 8.29 | N | Y | 140.00 | 38.50 | 19.64 | 5.84 | 1.95 | 1.17 | 3.78 | 1.18 | 1.27 | 57.4 | LPL(c.1187A>T) | - |
| 16 | M | 10.72 | N | Y | 150.00 | 48.80 | 21.69 | 6.14 | 2.57 | 0.96 | 4.01 | 1.26 | 1.22 | 241.5 | LDLR(c.1338del) | - |
| 17 | M | 12.99 | N | Y | 153.80 | 36.65 | 15.49 | 9.57 | 0.86 | 1.84 | 7.34 | 1.44 | 1.65 | 102 | LDLR(c.380T>A) | - |
| 18 | F | 4.39 | N | N | 100.70 | 16.60 | 16.37 | 9.04 | 0.68 | 2.01 | 6.72 | 1.59 | 1.73 | 11.1 | ABCG5(c.1673_1677del) | - |
| 19 | F | 8.51 | Y | Y | NA | NA | NA | 9.03 | 1.4 | 1.3 | 7.09 | 1.06 | 2.11 | 175.8 | ABCG5(c.1336C>T, c.1762+1G>A), LDLR(c.599T>G) | - |
| 20 | M | 2.88 | Y | N | NA | NA | NA | 10.36 | 0.53 | 1.5 | 8.62 | 1.39 | 2.36 | 40.9 | ABCG8(c.788G>A, c.1938_1939delins) | - |
| 21 | F | 2.55 | Y | Y | 89.50 | 12.20 | 15.23 | 7.28 | 0.73 | 1.51 | 5.44 | 1.55 | 1.55 | 4.60 | ABCG8(c.1256_1257delins) | - |
| 22 | F | 7.58 | N | Y | 127.50 | 36.70 | 22.58 | 8.52 | 2.27 | 1.86 | 5.63 | 1.66 | 1.64 | 115.40 | CETP(c.1103del), LDLR(c.1285G>A) | - |
| 23 | M | 4.71 | N | NA | NA | NA | NA | 8.59 | 1.63 | 1.16 | 6.69 | 1.23 | 1.81 | 8.90 | LDLR(c.2389G>A) | - |
| 24 | F | 5.30 | N | Y | NA | NA | NA | 7.27 | 1.27 | 1.00 | 5.69 | 0.82 | 1.65 | 100.80 | LDLR(c.327C>A) | - |
| 25 | M | 6.04 | Y | NA | NA | NA | NA | 15.65 | 0.77 | 0.66 | 14.20 | 0.61 | 3.73 | 20.10 | LDLR(c.817+1G>A, c.1298A>T) | - |
| 26 | F | 7.05 | N | Y | 128.70 | 36.00 | 21.73 | 9.22 | 2.05 | 1.10 | 7.19 | 1.23 | 1.94 | 91.30 | LDLR(c.1060+10G>A) | - |
| 27 | M | 11.81 | N | Y | 138.00 | 31.20 | 16.38 | 8.98 | 0.89 | 2.34 | 6.24 | 1.85 | 1.24 | 52.80 | LDLR(c.1187-10G>A) | - |
| 28 | M | 12.43 | N | Y | 161.00 | 71.50 | 27.58 | 6.16 | 3.00 | 1.22 | 3.60 | 1.40 | 1.10 | 27.10 | LIPC(c.748T>C, c.1052-1G>C) | acanthosis nigricans |
| 29 | F | 8.00 | N | NA | 136.00 | 27.20 | 14.71 | 7.61 | 1.14 | 1.50 | 5.59 | 1.36 | 1.53 | 120.60 | LDLR(c.905G>T) | - |
| 30 | M | 6.22 | N | Y | 115.00 | 19.60 | 14.82 | 7.71 | 1.13 | 1.35 | 5.85 | 1.28 | 1.51 | 35.70 | ABCG5(c.64C>T) | - |

^†^ All variants of genes were heterozygotes in this table.

F, female; M, male; SD, standard deviation; BMI, body mass index; TC, total cholesterol; TG, triglyceride; HDL-C, high-density lipoprotein cholesterol; LDL-C, low-density lipoprotein cholesterol; ApoA1, apolipoprotein A1; ApoB, apolipoprotein B; Lp (a), lipoprotein (a)
